# Supplementary material for: Fine scale prediction of ecological community composition using a two-step sequential Machine Learning ensemble
Source: PLoS Comput Biol. 2021 Dec 6;17(12):e1008906. doi: 10.1371/journal.pcbi.1008906 (PMC8675934; doi:10.1371/journal.pcbi.1008906)
Supplement: S1 Text — (PDF) [file pcbi.1008906.s001.pdf]

Fine scale prediction of ecological community composition using a two-step sequential machine learning ensemble (Supporting Information)

Icíar Civantos-Gómez[a,e], Javier García-Algarra[b], David García-Callejas[c], Oscar Godoy[d], Javier Galeano[e], Ignasi Bartomeus[f]

[a] *Universidad Pontificia Comillas, Faculty of Economics and Business Administration, Madrid, Spain*
[b] *Departament of Engineering, Centro Universitario U-tad, Las Rozas, Spain*
[c] *Departamento de Biología, Instituto Universitario de Investigación Marina (INMAR), Universidad de Cádiz, Cádiz, Spain*
[d] *Departamento de Biología, Instituto Universitario de Investigación Marina (INMAR), Universidad de Cádiz, Cádiz, Spain*
[e] *Complex Systems Group, Universidad Politécnica de Madrid, Madrid, Spain*
[f] *Estación Biológica de Doñana (EBD-CSIC), Sevilla, Spain*

S1. Abundance prediction with population dynamics models

Population dynamics models are arguably the main tool that ecologists use to do inference on ecological communities, from e.g. the effects of biotic interactions on populations to the expected long-term behaviour of a community. Here we show abundance estimates from the data set discussed in the main text, obtained with well established population dynamics models. We use the R package `cxr` (García-Callejas et al. 2020), which provides convenience functions to project community dynamics given information on 1) biotic interactions and vital rates, and 2) potential environmental covariates. In this file, given the annual nature of the system, we project a single temporal step and compare it with the observed values: for example, taking data from 2015 as a baseline, we project the abundances from 2016 and compare it to the observed ones. We repeat these projections for 2015, 2016, and 2018. The transition from 2017 to 2018 was left out because in 2018 there was a strong flooding event that wiped out most adult individuals from the study area, and this obviously cannot be accounted for with the population dynamics models we consider here.
We project the dynamics of the system with an annual plant model, a standard framework for projecting the dynamics of annual plants [1]. The model is of the form:

$$\frac{N_{i,t+1}}{N_{i,t}} = (1 - g_i)s_i + g_i F_i \tag{1}$$

$$F_i = \lambda_i e^{-\sum_{j=1}^{n}(\alpha_{i,j} N_j)} \tag{2}$$

where $N_{i,t}$ is the abundance of species i at time t, g_i is the seed germination rate, s_i is the seed survival rate, and F_i is the realized fecundity rate, which is a composite function. It is derived from the intrinsic fecundity rate in absence of interactions, λ_i, and the effect of biotic interactions $\alpha_{i,j}$ on it.
Note that, unlike data-driven models which only used observed abundances, this (or related) model need to be parameterized with coefficients that represent the factors expected to influence population dynamics: in this case, biotic interactions ($\alpha_{i,j}$), and vital rates (intrinsic growth rate λ_i, seed survival rate s_i, and seed germination rate g_i).
We generate two sets of predictions: in the first set, we only consider the effects of biotic drivers, e.g. species interactions and vital rates. In the second set, we use the insights from the two-step data-driven model to add important abiotic covariates. In particular, we add the spatial effect of carbonates to the expected species fecundity F_i:

$$F_i = \lambda_i (1 + \sum_{k=1}^{c} \theta_{i,k} c_k) e^{-\sum_{j=1}^{n}((\alpha_{i,j} + \sum_{k=1}^{c} \phi i,k c_k)) N_j)} \tag{3}$$

where $\theta_{i,k}$ is the effect of covariate k, in this case CO3, over λ_i, and $\phi_{i,k}$ is the effect of covariate k over every $\alpha_{i,j}$.
The error associated to the predictions from the population dynamics model is not directly comparable to the error associated to the data-driven model, because of the different methodologies and projections; this appendix is intended more as an example of how the insights from that model can help derive better mechanistic models. The inclusion of important abiotic drivers identified by the data-driven model significantly improves the error associated to the mechanistic predictions:

**Annual plant model**	**Median RSE** $(1 - R^2)$	**Median RMSE**
biotic	4.30	53.32
$biotic + CO3$	1.70	13.26

Table A: **Prediction errors for annual plant models with and without the inclusion of CO3 as an abiotic driver.**

These models also show a high variability among species, with a few species having extremely high error rates in comparison to the rest (Figure similar to Fig. 3 of main text).

S2. Supplementary figures

Exploratory analysis

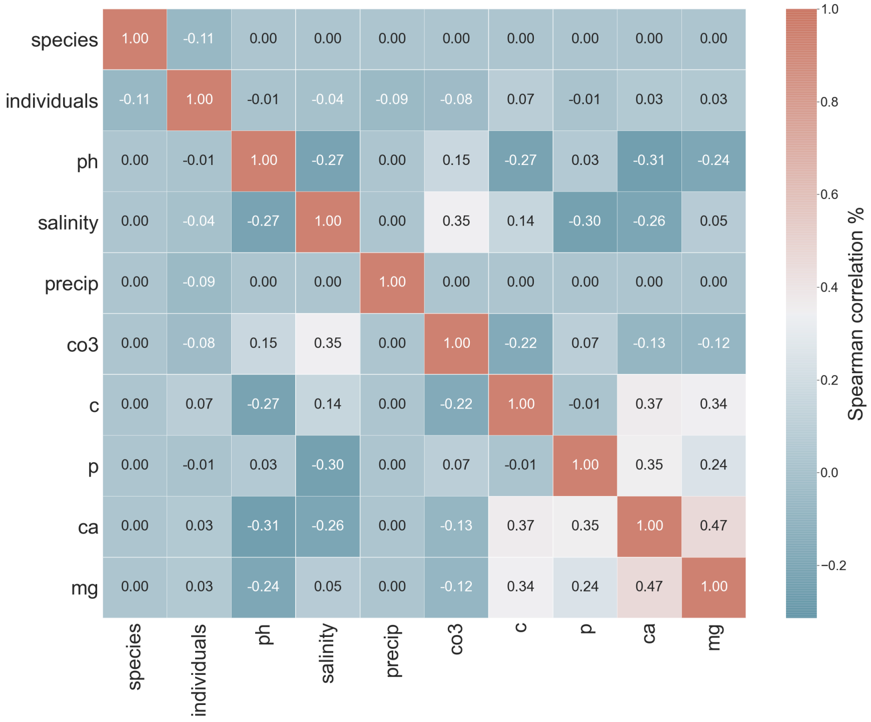

Figure A: **Abiotic features correlation.**

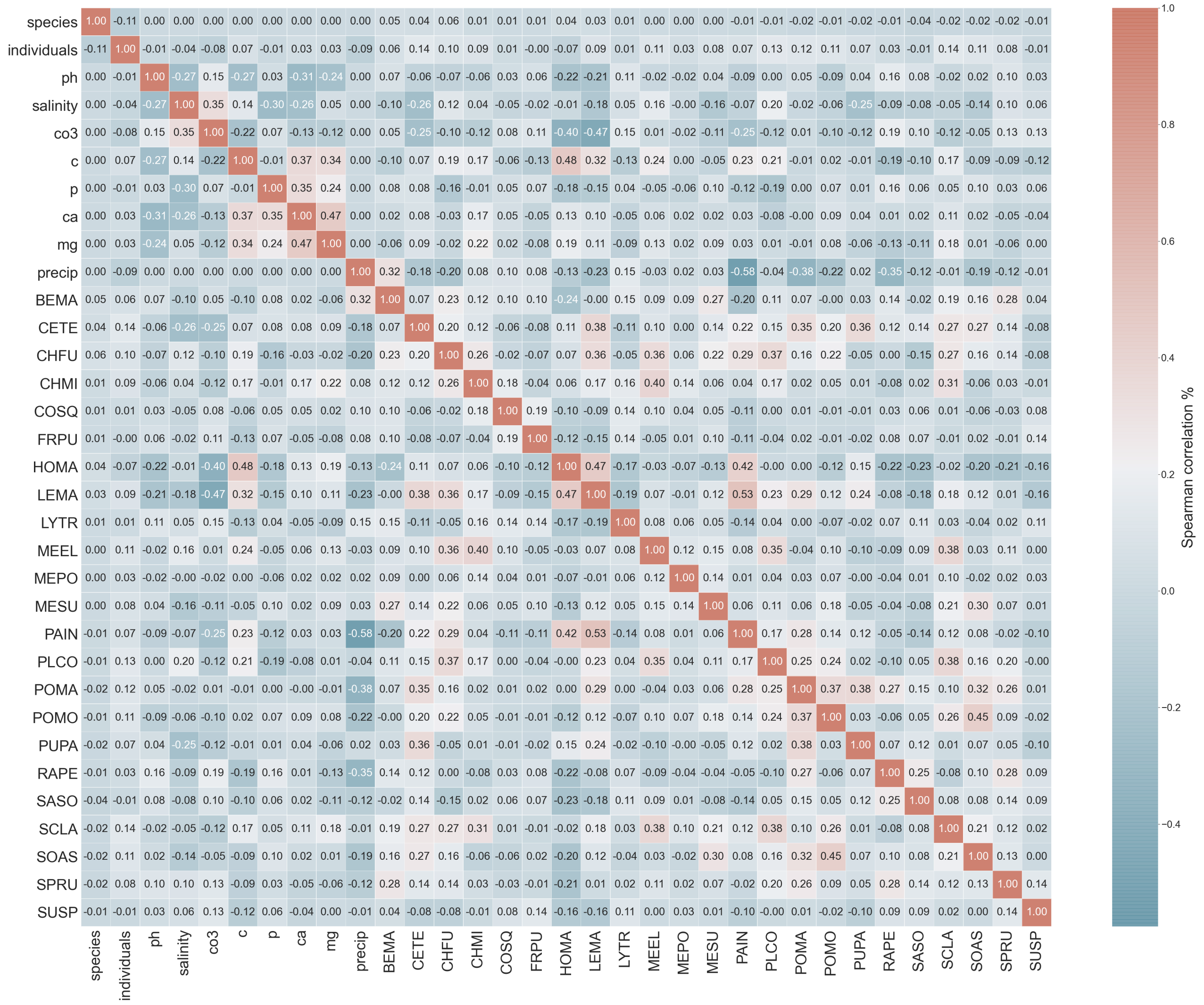

Figure B: **All features correlation.**

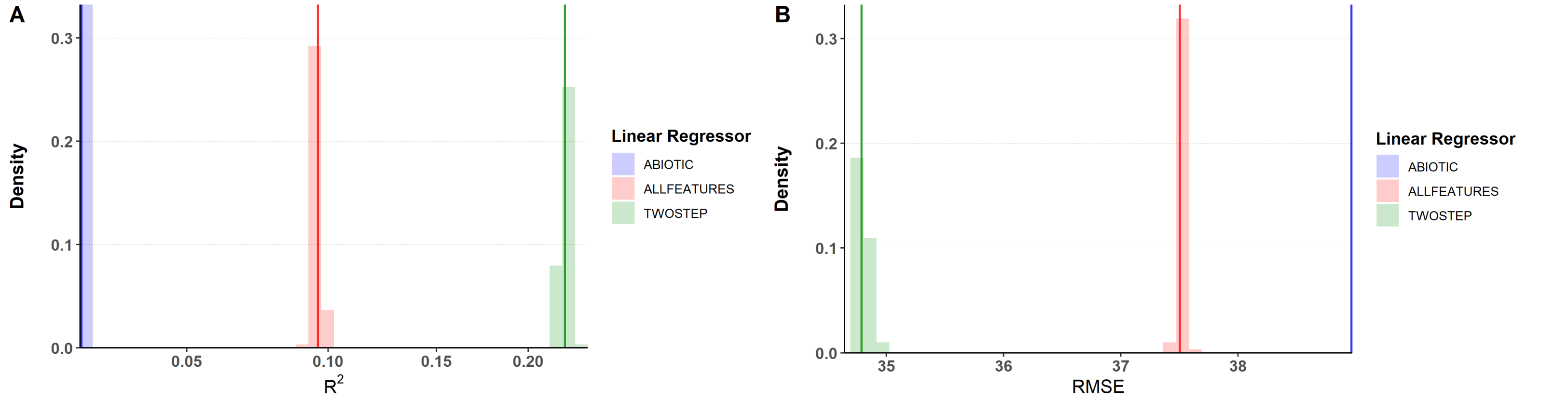

Figure C: **Prediction errors with a Linear Regressor.** A: R^2 distributions for 100 random choices of training/testing sets, vertical lines set at median values. B: Root Mean Square Error distributions for the same collection of predictors.

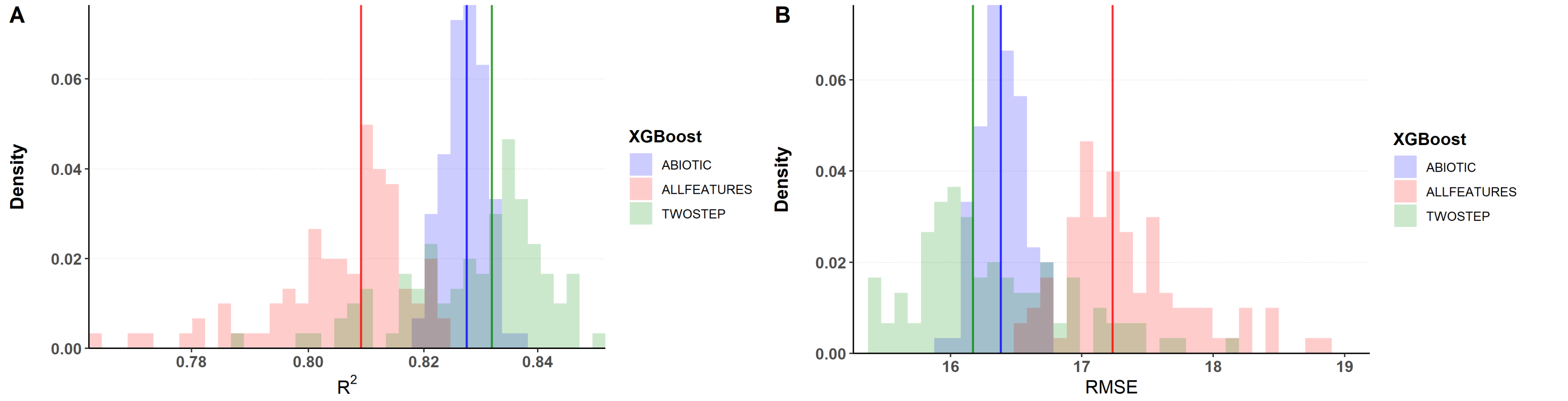

Figure D: **Prediction errors with a XGBoost Regressor.** A: R^2 distributions for 100 random choices of training/testing sets, vertical lines set at median values. B: Root Mean Square Error distributions for the same collection of predictors.

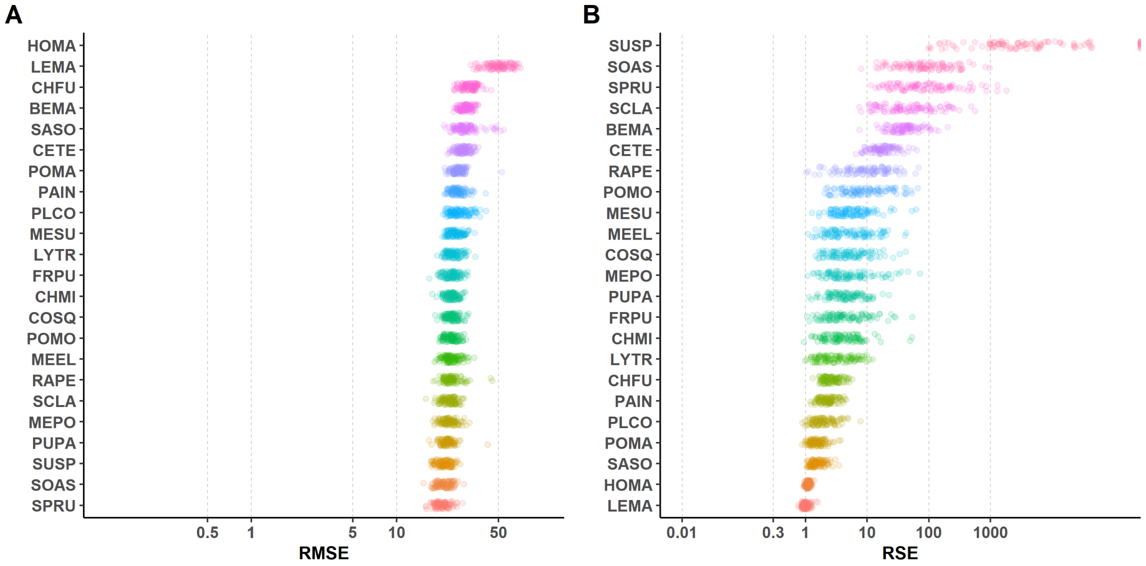

Figure E: **Prediction errors by species using a Linear Regressor.** A: Relative Squared Error distributions for 100 random choices of training/testing sets. B: Root Mean Square Error distributions for the same collection of predictors.

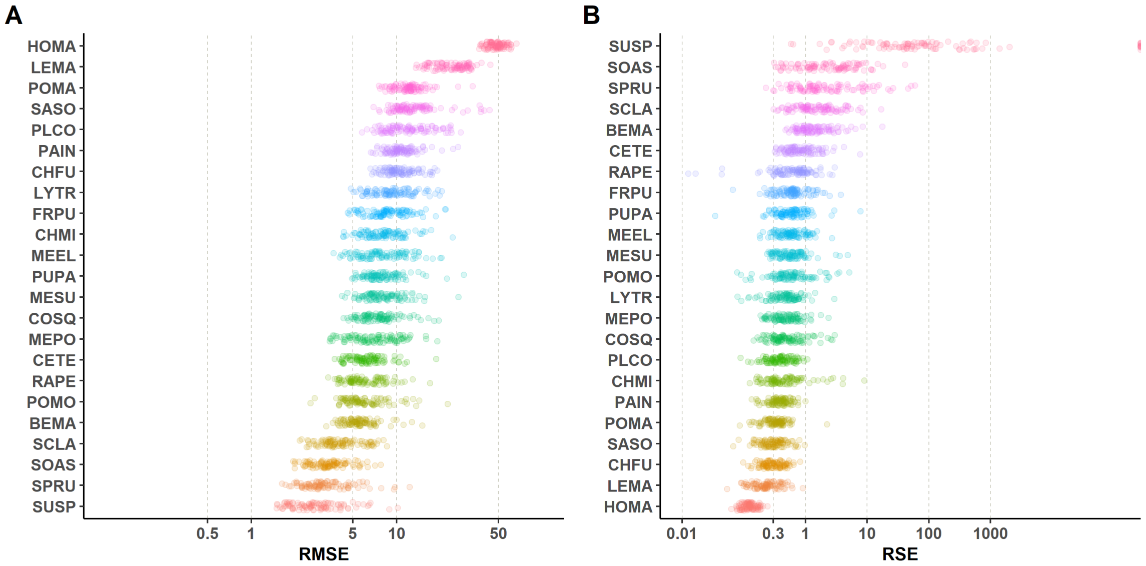

Figure F: **Prediction errors by species using a XGBoost Regressor.** A: Relative Squared Error distributions for 100 random choices of training/testing sets. B: Root Mean Square Error distributions for the same collection of predictors.

S3. Supplementary tables

Table B: **Abiotic variables** (1/5 means one part of soil divided in five parts of water)

Variable	Data field	Units
Annual precipitation	precip	mm
pH	ph	$pH1/5$
Salinity	salinity	$Ce\ 1/5\ dS/m$
Chlorine	cl	$Cl^{-}\ 1/5\ mg/l$
Carbonates	co3	$CO3$ %
Carbon	c	$\%\ C\ oxi.$
Organic matter	mo	mg/l
Nitrogen	n	$Nitrogen\ [NKjeldahl(\%)]$
C/N ratio	cn	–
Phosphorus	p	$Olsen\ P\ mg/kg\ soil$
Calcium	ca	$Ca\ Assimil.\ mg/kg\ soil$
Magnesium	mg	$Mg\ Assimil.\ mg/kg\ soil$
Potasium	k	$K\ Assimil.\ mg/kg\ soil$
Sodium	na	$Na\ Assimil.\ mg/kg\ soil$

Table C: **Plant species**

Species	Data field
Beta macrocarpa	BEMA
Centaurium tenuiflorum	CETE
Chamaemelum fuscatum	CHFU
Chamaemelumm mixtum	CHMI
Coronopus squamatus	COSQ
Frankenia pulverulenta	FRPU
Hordeum marinum	HOMA
Leontodon maroccanus	LEMA
Lythrum tribracteatum	LYTR
Melilotus elegans	MEEL
Medicago polymorpha	MEPO
Melilotus sulcatus	MESU
Parapholis incurva	PAIN
Plantago coronopus	PLCO
Polypogon maritimus	POMA
Polypogon monspeliensis	POMO
Pulicaria paludosa	PUPA
Ranunculus peltatus	RAPE
Salsola soda	SASO
Scorzonera laciniata	SCLA
Sonchus asper	SOAS
Spergularia rubra	SPRU
Suaeda splendens	SUSP

Table D: **Feature importance for the Random Forest predictor with all variables**

Feature	Importance
PAIN	0.2180
Species	0.1669
LEMA	0.0826
Annual precipitation	0.0721
CHFU	0.0643
C	0.051
POMA	0.0457
Carbonates	0.0315
HOMA	0.0285
Salinity	0.0269
PUPA	0.0254
P	0.0219
Ca	0.0210
random noise	0.0189
PLCO	0.0174
SASO	0.0149
mg	0.0134
ph	0.0127
MESU	0.0109
CETE	0.0102
RAPE	0.0087
BEMA	0.0048
SOAS	0.0045
CHMI	0.0045
POMO	0.0042
MEEL	0.0027
SPRU	0.0026
SCLA	0.0026
LYTR	0.0024
SUSP	0.0004
COSQ	0.0003
MEPO	0.0002
FRPU	0.0001

Table E: **Linear regression coefficients with the abiotic set**

Feature	coef	std err	t	P>|t|	[0.025	0.975]
Species	-0.5996	0.030	-19.735	0.000	-0.659	-0.540
Salinity	1.0240	0.160	6.414	0.000	0.711	1.337
Annual precipitation	-0.0138	0.002	-6.339	0.000	-0.018	-0.010
Carbonates	-0.4434	0.120	-3.688	0.000	-0.679	-0.208
C	6.2165	0.681	9.126	0.000	4.881	7.552
P	-0.2185	0.083	-2.629	0.009	-0.381	-0.056
Ca	0.0043	0.000	11.852	0.000	0.004	0.005

References

[1] Chesson PL. Geometry, heterogeneity and competition in variable environments. Philosophical Transactions of the Royal Society of London Series B: Biological Sciences. 1990;330(1257):165–173.
