# Supplementary material for: Fine scale prediction of ecological community composition using a two-step sequential Machine Learning ensemble
Source: PLoS Comput Biol. 2021 Dec 6;17(12):e1008906. doi: 10.1371/journal.pcbi.1008906 (PMC8675934; doi:10.1371/journal.pcbi.1008906)
Supplement: Attachment — Submitted filename: Report.pdf [file pcbi.1008906.s003.pdf]

Referee Report

Great paper, well written, nice plots, strong methods. So, I don't have much to suggest but some edits to make it more readable.

General remarks

- I wonder how much the two step model performance depends on the strength of the interaction. One way of understanding this, would be to compare the expected size of the population in isolation vs accounting for interactions. How quickly the method would stop to be accurate if the interactions increases?
- How would you account for other kind of ecological interactions (plant-pollinator, plant-herbivore, ...)? If we are considering the method in the framework of climate change adaption, may the impact of climate change on other guilds of the ecosystem be an even stronger force than abiotic ones?
- It is not clear to me the dependency from the spatial and temporal model. In particular, I can't understand how the "latter" temporal model is different from the spatial model. It may be worth to explicit precisely what data is used at each step of training and testing for each model.

Section wise remarks

Abstract

I would not show RSE here as, without a context, it doesn't mean much (at least to me: it's a big number? a small number? what would be a big number?).

Section: The Problem

The motivation for the paper is clearly expressed, however the mathematical formalisation of it is unnecessary heavy.

Taking for example Eqn (1), I would suggest to rewrite it as:

$$\tilde{X}\_j\left(t\right) = g\left(A\_1\left(t\right), \ldots, A\_n\left(t\right), X\_1\left(t\right), \ldots, X\_n\left(t\right)\right)$$

That is: the term $X\_2\left(t\right)$ is unnecessary and the dependency on the subplot k can be stated once and for all (and then drop from notation). This holds for all the equations in this section.

In Eqn. (2) the subplot becomes m instead of k. I couldn't understand why, and it conflicts with the m stating the number of species. In Eqn. (5) the subplot becomes p. Keep k everywhere (and keep it implicit for a lighter notation).

In Eqn. (5) I would consider using $\tilde{g}$ instead of f as f has the same arguments and outputs of g and it is indeed the task of the machine learning models to approximate it. This also makes clear that there are two approximation steps, one for $\tilde{X}$ and one for $\tilde{g}$.

Also, when introducing the two step spatial model (based on Eqn. (5)) I got at first confused about the temporality of the events being predicted at each step. My current understanding is that: - Step (1): from the $A(t-1)$ and $X(t-1)$ predict $A\_pred(t)$ and $\tilde{X}(t)$ (the abundances if there were no other species in the plot) - Step (2): from $A\_pred(t)$ and $\tilde{X}(t)$ predict "biotic constrained abundances" $X(t)$ If that is the case, and I'm not confused, it may be worth to explicit it.

- line 111: I would use the term 'subplot' instead of 'area' as the term 'area' is not used again throughout the text

Section: Methods

- line 180: I would drop "Therefore"
- line 192: "black models" -> "black box models"

Section: Results

- lines 294 - 297: I'm not sure I would say that "machine learning" methods for time series prediction require, in general, a trend and a seasonality. Any machine learning method should, at least, predict quite well a constant times series (one that never changes value). Either or both of those things can be missing, and prediction can still be made. Yet, I may be misunderstanding what you are saying here.
- lines 256 - 259: Maybe explicitly formulating the models for the spatial and temporal predictors might clarify the distinction between the 2 models for readers specially for the step 2? (It could be included in the supplementary information)

Section: Appendixes

Results from S1 sounds like some interesting results, would it be worth mentioning in the main body?
