# Supplementary material for: Fine scale prediction of ecological community composition using a two-step sequential Machine Learning ensemble
Source: PLoS Comput Biol. 2021 Dec 6;17(12):e1008906. doi: 10.1371/journal.pcbi.1008906 (PMC8675934; doi:10.1371/journal.pcbi.1008906)
Supplement: Attachment — Submitted filename: response_to_reviewers.pdf [file pcbi.1008906.s004.pdf]

REVIEWER #1

#	Remark
1	Spatial correlation and sample size: How is spatial autocorrelation among the 36 subplots accounted for? What about the 9 plots (which are all < 1km apart). Abiotic properties are measured at the scale of the plot not subplot (line 158) so how is this not pseudoreplication? We really need to see a variogram or correlogram showing a lack of spatial autocorrelation (which, honestly, is a bit unfathomable) otherwise you need info about how spatial correlation structure was included in your models. In addition, if you are relying on ensemble resampling (in fitting) and cross-validation (in validation) is absolutely critical that you do so blockwise (i.e. dropping whole sites) otherwise your error statistics are going to be massively optimistic.
	Thank you for pointing this out. We are aware that ecological data often show spatial structure. Cross-validation scores for spatial data can be biased because observations are commonly spatially autocorrelated (closer data points have similar values). In other words, when performing cross-validation, these structures are regularly ignored, resulting in serious underestimation of predictive error. One cause for the poor performance of uncorrected (random) cross-validation, noted often by modellers, are dependency structures in the data that persist as dependency structures in model residuals, violating the assumption of independence. We now use Block cross-validation, where data are split strategically rather than randomly, and is suggested to be a good approach to address these issues. In particular, when splitting the data into training and testing sets, BlockKFold first splits the data into spatial blocks and then splits the blocks into folds. During k-fold cross-validation, one fold is used as the testing set while the rest are used for training. Since each block can have a different number of data points, assigning the same number of blocks to each fold can lead to folds with very different numbers of data points. By default, BlockKFold takes care to balance the folds to have approximately equal number of data points. The results... In addition, note that most measures are taken at the subplot level (salinity, number of neigbougs, ...) and that the study system presents a high microscale heterogenetity as described in methods.<br>We check for the spatial autocorrelation of each species abundance and found that for all species the Moran I was low ($I < 0.2$). This is because the high spatial heterogeneity caused by the interplay between the microtopographic changes and the high salinity. For example, subplots 1 meter apart often show changes in their salinity values that exceed those separated by thousans of meters. Hence, we do not further model the spatial component directly in our models, but we do take into account the spatial distribution in our training and testing sets
2	Zero inflation: The dataset being analyzed contains 75% zeros, but the authors don't really explain how they handle the distinction between presence/absence and abundance. They are working with models that don't have built-in range restrictions (abundance can't be negative), nor do they account for the discrete nature of abundance data, but they say nothing about how this affects prediction (e.g. models could easily predict non-sensical negative values, but we don't hear/see anything about how often this occurs or how it's handled; see Model Performance below) or scoring (are RMSE and RSE really the right metrics for scoring zero-inflated count data?)
	We add an explanation defining when zero means in our dataset: "Each of them [zero avlues] means that the field team has not found any individual of the particular species inside the sampled subplot during the season". The reviewer is righ that the chances of predicting negative values are not null, and now we include the magnitude of this effect in the Results section. See below for the RMSE/RSE discussion).<br>About negative values, please see remark #35.
3	Model selection: Counter to the basic concept of parsimony and decades of literature on model selection (plus just common sense), the authors retained covariates that contributed less to the model than random noise. Similarly, covariates were included in the model that were massively colinear, counter to what anyone would learn in a basic stats course, even when it's obvious a priori that these are not telling us different things (e.g. including all of C, N, C:N, and organic matter; including all of sodium, chloride, and salinity).

We reworded for clarity and remove some variables to re-assure our findings are robust: "Decision tree methods, in particular random forests and boost decission trees, and ridge regression are not much affected by multi-collinearity [citation Dormann, Elith et al 2012]. However, since it is a good practice to remove any redundant features from any dataset used for training, we used Spearman correlation as a filter-based feature selection method. By using this technique, features such as Cn, Mo and N were discarded for model training. Nevertheless, performance was not affected in a sensible way by removing such variables."

4	Model performance: There are no plots showing how the model actually does at predicting abundances, just plots of error statistics. Similarly, there are no plots of what the inferred relationships between inputs and abundance actually look like, which severely limits interpretability and greatly reduces what we're learning from the analysis. There's not even information on a species-by-species basis about the relative importance of the different abiotic covariates and where different species are sorting out on the abiotic gradient. Similarly, there's no information anywhere in the paper about what the ML model tells us about how the species are interacting (just a correlation matrix of the RAW data)
	Thank you for this comment. Our main aim is to show overall performance, which is accomplished with the error statistic plots.We have added Fig. 4 to show the distribution of individual prediction errors for a particular run out of 100, of the two-step RF and the abiotic RF models.
5	Strawman comparison: In the paper the authors compare their ML models to predictions made by a linear regression. I think this is a strawman comparison because conventional analyses for community composition data have for many decades acknowledged that the relationships between predictors and abundance are nonlinear (e.g. Canonical Correspondence Analysis assumes a unimodal Gaussian kernel). In showing they do better than a regression, and then identifying the relative importance of a few abiotic variables, I don't think the authors demonstrate that they learned something that wouldn't have come out of conventional vegetation analyses.
	We are sorry if the linear regression looked like a strawman comparision. We knew it is an unfair and artificial comparision, and we showed it only to show that the two-step approach can be implemented with any modeling technique. We clarified this in text. Our aim is not to compare model performance across different modelling techniques, which is beyond the scope of the paper.
6	Temporal model: Temporal model is poorly described, and in particular how spatial information is leveraged when making temporal predictions. Are the sensitivities assumed to be the same? That seems to be implied, and a space-for-time substitution may be OK as an initial hypothesis (in which case you may not need longer time series), but there's also lots of evidence that responses to mean climate and responses to temporal anomalies need to be treated differently (though the two may interact).
	The section "The problem" has been rewritten to explain the differences of spatial and temporal applications of the predictive model and clarifies that we have a single model, but with different validations (temporal vs spatial).
7	Paper has numerous spelling and grammar errors; would really benefit from a copy editor. Mathematical notation is inconsistent.
	Reviewer #3 has pointed the mathematical notation issue as well. Please, find how we have fix it in the answers to "Problem 2" and "Problem3", "Reviewer 3" Tab.
8	The paper has new analyses showing up in the Results and Discussion that weren't motivated or explained in the Introduction and Methods (e.g. fitting a simple demographic model), as well as analyses in the Methods that don't have Results or Discussion (e.g. Taylors Law).
	The observation of how data fit Taylor's Law is just a product of the exploratory analysis. We add this paragraph in Methods section: As initial data assessment, we performed an exploratory analysis, studying abundances distribution for each species and their relationship between averages and variances.

9	Abstract line 9: I don’t think you benefit from having the word “deterministic” here, it’s just going to be a distracting source of contention (immediately caused the reader to start debating whether ecological processes are deterministic, which is beside the point [especially if they are predictable, which isn’t the same thing as deterministic])
	"informed by ecological deterministic processes" removed form abstract
10	Abstract line 14: RSE referenced for the first time without defining the acronym. This metric isn’t particularly common, so you can’t assume meaning from context, nor is it clear from context whether 0.13 is a good score). You might try using R2 instead, which is more commonly understood.
	As suggested we switch from RSE to R2, except for the plot "Prediction by species" where a log representation is more convenient and we keep the RSE. We drop the RSE value of the abstract.
11	Abstract line 19: not clear what “back” refers to here<br>Abstract line 22: probably need a “predicting” between “at fine-scale”, as well as a comma before the “while” and the “can” on the following line.<br>Abstract line 23: especially, not specially
	19. Reworded "can shed light on data driven models"<br>22. Reworded "Being able to build improved models at predicting fine-scale species composition, while ..."
12	Author summary line 8: need a “by” in “abundances training”<br>Line 2: drop “different drivers of”<br>Line 8: “experienced fast growth during the last decades due to”<br>Line 27: missing a word in “require to feed” (e.g. require data to feed)
	Reworded as suggested
13	Line 46: the statement “high level of abstraction of mechanistic models” seems like an oxymoron to me (if it’s mechanistic, it’s not an abstraction). More generally, throughout this discussion you need to be much more clear about what you consider a "mechanistic model". Many of the statements you make apply to simple theoretical models (e.g. Lotka-Volterra), but I would not consider such models to be mechanistic, especially compared to the many plant community and population modeling approaches that explicitly account for demographic processes (e.g. matrix models, integral-projection models, individual based models, stochastic simulators like forest gap models) or ecophysiologically-based vegetation demographic models (see review by Fisher et al 2018 GCB). It is worth noting that while many of these approaches do have large numbers of parameters per species (or PFT), many also model resource competition explicitly (rather than having theoretical “interaction terms” and thus the number of parameters scales linearly with the number of species in the community).
	Thanks for pointing that out, we clarified this sentence to include the complexity of different modelling approaches.
14	Line 99: you should NOT include “individuals of Xj(k,t)” as a covariate when trying to predict the abundance of species j. Such a model is circular and trivial. The “including” should be “excluding”.
	The remark is quite important, and the covariate of individuals of the same species must be excluded. As the training set includes samples of any possible species at the focal point of the subplot ("individuals" feauture), all covariates are used to build the predictor and columns can't be droped. To avoid this issue, we used a zero abundance for the species of the focal point in the original dataset. This is a reasonable workaround, that excludes the effects of instrasepecif competition, but breaks the circular dependence. For the two-step model, this is not an issue.
15	Line 115: no clue what “abundance of the plants that will ripe next year” is referring to. You’re discussing models of abundance, not maturation or fecundity.
	End of the sentence removed, because the model doesn't deal with fecundity, as the remark underlines.

16	Eqn 3: The model presented here is subtlety but importantly different from conventional time-series and dynamic modelling approaches to predicting temporal variability, which would use the abundance of the con- and heterospecific individuals at time t (not t+1) to make predictions to time t+1. The whole problem described in the rest of the paragraph changes dramatically when you do this. Yes, you still are faced with the problem of needing to know the current state of the vegetation (a.k.a initial conditions) at new locations, but that's the same problem as your spatial prediction problem (not a new problem), and also one that is more constrainable by data than the "unavailable by definition" problem of knowing the abundances in the future. At the same time you may genuinely want/need to include the covariates at t+1, and yes those need to be predicted, but this is a standard ecological forecasting problem and not one that needs to be built into your ML model (e.g., using the long-range seasonal precipitation outlook to predict species responses to interannual climate variability). Finally, when making community-level predictions you wouldn't be writing a separate model for each species, but would be writing a multivariate model that predicts a vector of abundances and accounts for the covariances across species.
	The section "The problem" has been rewritten to make clear that the model is just a conventional supervised predictive model with spatial or temporal applications. The predictor is unique for all species.
17	Line 126: I can't figure out how you would end up with 23 unsolvable problems when you have 20 competitors.
	That sentence was a typo and has been removed. The paragraph has been slightly reworded to better explain the recursive nature of the prediction problem
18	Line 148: Is "Prediction" the best title for this subsection? Seems like it's mostly a description of the sites and data
	Fixed, changed to "Data description"
19	Line 149-157: This is insufficient information about the field research. Where exactly were these plots (lat/lon? Country? Field station?). How exactly where the communities surveyed? How many soil cores were taken per plot? How deep? What methods/protocols were used to analyze the soil properties? What equipment was used? What type of system measured precipitation?
	We added the requested information.
20	Line 165: When you say "Removing them", what specifically are you removing? Need more info about the specific criteria you used to remove species or sites, whether that was for the whole analysis or just a specific plot, and how sensitive you results are to your removal criteria. In general, subjective criteria for dropping datapoints makes me nervous.
	Sorry, it really was a puzzling sentence because we were trying to describe the effect of a hypotethical removal of samples in the overall distribution of abundances. We reworded it as: " Even if zero values were removed, the uneven distribution of abundances would remain, as generally expected from species-abundance distributions". At the end of the paragraph we append: "In any case, no sample is discarded to build the predictors."
21	Line 167-171 and Fig 1B: Not sure why there's this text and analysis about Taylor's law. This doesn't seem to be motivated by any question posed in the study (Intro), nor does it show up again in the Results or Discussion, it's just a random analysis thrown in for no apparent reason. I'd recommend dropping.
	See remark #8.
22	Line 182: "generally works fine" feels like a subjective value judgement. Not sure why it's here (rather than Discussion) nor why it's relevant.
	The expression has been removed
23	Line 198: If you are fitting a model with 40 covariates to a dataset that contains 5 years of data for 9 sites (45 site years) how is this not overfitting?
	The basic sampling unit is the subplot. There are 36 subplots for each ofthe 9 plots, and the series spans 5 years, so there are 1620 sites/year. We have improved the Data description subsection

24	Line 200: organic matter<br>Line 204: what does “tried to” mean? Did the implementation fail somehow?
	200.Fixed<br>204.Reworded, a translation problem
25	Line 208-212: First, the descriptions of these three methods is really insufficient to follow what is being discussed. Second, if you used the Filter method, why are you even discussing the Wrapper and Hybrid? No need to describe methods you didn’t use.
	References to Wrapper and hybrid methods removed
26	Line 213: First, the discussion of correlations seems like it’s a completely new topic, and thus should be a new paragraph (if it’s not a new topic, it’s unclear how it’s related to the filter method. Second, what is your threshold for “significant”. Third, how is it possible that there were no significant dependencies? The correlation matrix shows that C and OM have a correlation of 1! C also has a 0.93 correlation with n and 0.97 with C:N. How could these not be significant? Similar story with the correlations among salinity, Na, and Cl (not surprising). You absolutely should have reduced the dimensionality of these data before starting any analysis to avoid spurious results.
	Organic matter, Cn, Na and Cl have been removed to build all models. We explain the details in methods.
27	Line 215: This test is being introduced without any transition or explanation of what this is or why this is being applied.
	The Permutation Importance test has been applied to remove features with low predictive power. We improved the original wording for clarity.
28	Line 216: First, this is Results not Methods. Second, tell the reader which 25% was important (don’t make me stop and go find and interpret your table). Third and most important, were the other 75% of the variables dropped?<br>Line 220: this is also Results not Methods.
	Apologizes for the confusion, the 25% figure was not even correct. We have rewritten this paragraph and moved the results of the correlation analysis and feature Importance from Methods to Results, as suggested.
29	Line 223: The decision to retain variables that add less to your model than random noise makes absolutely no sense and is a pretty textbook example of what not to do in data analysis. Further, the argument that dropping the variables doesn’t improve predictions also completely misses the basic idea of parsimony and the core concepts underlying model selection. If we take the linear model as an example, every additional variable you retain increases the parameter uncertainty and thus increases the predictive variance. This is only worth doing if the residual error goes down by more than the parameter error goes up, such that the net predictive variance declines. Given that 75% of your variables are no better than random, adding them is clearly not improving the model, so they cause a net increase in predictive variance (which we’d see if the authors accounted for predictive uncertainty, which they don’t seem to be doing). But even adding a random variable will cause the residual error to at worst stay the same, and sometimes go down a bit by chance, so I have no idea where this “removal makes the model better” criteria is coming from.
	We apologize for the messy description of the procedure and the selection criteria. We hope that the new organization of the text makes more clear our point.
30	Table 1: (1) Given that you described three modeling approaches, it’s unclear which model is being reported in this table. (2) what does “species” represent in the abiotic model? This factor wasn’t ever described in the Methods. (3) I’m assuming that this model is averaging the importance over all 23 species that were modeled, which seems like it could be really misleading, as we’re not learning anything about which species are associated with which drivers. (4) If C and OM have a correlation of 1, how come their importance scores are so different (with one being “significant” and the other being worse than random noise)

(1) Caption changed to "Feature importance for the Random Forest model with the ABIOTIC set of variables"
(2) We have added this sentence at the end of the subsection "Feature engineering" in Methods: 'There is also a factor called species that is the acronym of the plant species at the focal point of the subplot. We will build a unique model that works for any focal species, so this factor must be kept to inform the predictor'
(3) As the reviewer points the model is unique for the 23 species and that may be a burden for interpretability. On the other hand, building individual models for each species may be unpractical if we want to extend the analysis to other areas of the National Park accounting for dozens of additional species. That was the reason why we decided to keep it as simple as it was possible.
(4) The Feature Importance method is sensitive to collinearity as it builds the model before evaluating the weight of each feature. The proper procedure is removing collinear variables before running the Feature Importance method, and that is what we have fixed and explained, following reviewer's suggestion.

31 Line 228: Please don't change your notation in the middle of the Methods as it's really confusing. Up to now Xi were the observed values and \hat{Xi} was the model predictions. Now you've switch y for X for no reason. Also, much more confusing, you're now using the hat for observations and the non-hat for predictions, which is the EXACT OPPOSITE of your previous notation.

It was a typo, fixed.

32 Eqn 7: is there a reason to use RSE over the much more commonly used R2 (which should just be 1-RSE)

Following reviewers'suggestions we switch to R2

33 Line 257: see previous comment about the need to block your training and cross-validation rather than subsampling randomly. See for example Roberts et al., 2017 "Cross-Validation Strategies for Data with Temporal, Spatial, Hierarchical, or Phylogenetic Structure." Ecography.

The cross-validation method has been changed following the suggestion. "Then, for each model we perform a 4-fold spatial cross-validation [roberts2017cross] using the K-Folds cross-validator provided by the Python package Verde"

34 Line 252-253: First, it's unclear why you would want to "avoid" the zeros in the data – the data is what the data is, and there's no reason the expect absences to be "wrong" in any way. You should model the data appropriately (as zeros) rather than trying to transform your absences into something else. Second, the transformation you applied wasn't described at all, you need to add a lot more detail. Third, given that the data is non-negative but you aren't using distributions or models that enforce non-negative predictions, you also need to explain how you're handling negative predictions from your models. Given a ~75% zero rate, I can only assume that there are a LOT of negative predictions. Fourth, while we're on the topic, there's not a single plot of what your model actually predicts in the whole paper (e.g. a predicted/observed plot, or plots of predicted abundance as a function of location, time, or your dominant covariates); how can a reader assess how your model is doing if we never get to see what it's predicting?

This paragraph was rather confusing. Zeros were never removed and the reference to SMOTE was useless, as the procedure was an initial try, and it should have been deleted from the code. We have done it now, and the whole paragraph is removed.

35 General Methods comments: (1) What tools were used to do all of this? What versions of what packages and where are the citations? Right now none of these is reproducible. (2) your Methods never really explains how you're handling the discrete nature of your count data, the zero inflation, or negative predictions. (3) given that you're arguing for a sequential approach to modeling, it seems like you really need to show that your approach outperforms fitting the community data all at once (rather than sequentially).

(1) Package list and citations have been included at the end of the document
(2) The Random Forest models do not predict negative values. The Linear Regressor and XGBoost, return between 9% and 25% of negative values that would not have biological sense. We have kept them as predicted to compute the R^2 index, and we have kept the decimal values as well, in order to make fair comparisons among the different models.
(3) Fitting all at once is what we have called the ALL FEATURES model, we do apologize if the original explanation was not clear enough

36 Line 265: You should not be introducing new Methods details in the Results.

Fixed, the sentence has been moved to the Methods section

37 I think you're misinterpreting RSE, which is the % better than a simple null mean model, not the % error (which most readers will interpret as a something like a coefficient of variation)

Fixed, when introducing the measure (remark #32)

38 Line 278: Three really important, bit points:
(1) There needs to be more information in the Discussion about WHY this sequential method improves prediction as what's going on here is a bit too opaque and unintuitive and the explanations being given are too hand-wavy. To give a concrete example, if express the first abiotic model as \tilde{X} = alpha A (where alpha is a vector of coefficients and A is the matrix of covariates) and the second as X = beta A + gamma \tilda{X}, then you could just substitute \tilda{X} in to get X = beta A + gamma alpha A and it's not clear (a) how this could possibly do better than just beta A [seems like it should be identical], (b) how it's not overparameterized, and (c) where the additional information content is coming from to be able to improve predictions.

The new analysis shows that the sequential no longer improves predictions, but it matches the predictions of the full model. We change this paragraph accordingly.

39 (2) If there's such a large improvement over a basic linear regression, that implies that key predictors are nonlinear. Please visualize those relationships so we can see if what the model is doing is sensible / biologically plausible or whether it's overfitting. This is also the ecologically interesting/important part of the model. Telling me that plants associate with soils and precip it trivial. Telling me what that relationship looks like lets me think much more deeply about what processes are at play here.

The improvement is not so dramatic, after changing the cross-validation method. The results of the two-step model are almost identical to the all features model (Table 2 & Fig 2). The improvement may be interpreted as the information added by the competitors abundances.
To understand why the performance of both models is so different, we compare the importance of features of LR and RF using the ABIOTIC set of variables. We include two additional tables in the Supporting Information and add this paragraph in the Results section of the draft.
"The weak performance of the Linear Regressor is a hint on the non-linear nature of the prediction challenge. The F statistic for the abiotic data set trained LR, is nearly null. According to the t value, the order of significance of variables is Ca, C and salinity, with the annual precipitation in fourth place (Supplementary table S5). Even though is a rough way to compare, the Feature Importance for the RF model is quite different, with the annual precipitation as the most important variable. "

40 (3) Competing two ML models against a linear regression seems to be a bit of a straw-man argument. The long-standing textbook expectation for vegetation abundance along a gradient is that these patterns tend to be unimodal (e.g. classic papers by Whittaker from the 1950s). If abundance is unimodal, then the best-fit line will often be a pretty flat line (hence the trivial improvement over a null). This is also why things like Canonical Correspondence Analysis (which assumes a Gaussian abundance kernel) have been standard practice in community ecology for decades. This also reiterates my second point (we all already knew these relationships were nonlinear, but can you show that we really need something different from a Gaussian?)

The idea of the paper was not comparing methods. We clarified this in text.

41	Line 295: WHY would this be a required condition (seasonal variability and long term trend), especially if what your temporal model is trying to predict is interannual variability, not seasonal or trends?
	Sentence removed, as the reviewer suggests it was pointless in this scenario
42	Line 309-311: This point about variability falling outside previous observations reinforces my previous question about a lack of clarity of how spatial and temporal variability are being handled in these models. It seems less likely that the observed temporal variability fall outside the range of both the spatial gradient (n=9) and the interannual variability (n=5) simultaneously.
	We hope that the explanation on how the model is applied to spatial and temporal applications and the improvement in the description of the data set makes clear this point.
43	Line 312: I'm a bit concerned about the idea of dropping what is clearly the most important predictive variable (precip, table 1) unless you've also already dropped all the other less important variables (in which case you're just fitting a constant mean)
	The idea here is that precipitation is a proxy of the year, as the recorded value is unique for season. So, including precipitacion when splitting in training/test by year is a source of overfitting. Anyway, the results are extremely poor even with this workaround and our conclussion is that the series is too short (five years) to build a working predictor for incoming seasons.
44	Line 316-17: Be much more specific about what novel insights are coming out of these models. This needs to be something much more impressive than "precipitation structures communities" or "carbonates matter", as this is too vague and also something that I suspect would have come out of traditional vegetation community analysis techniques (e.g. CCA)
	We re-worded the paragraph to indicate that identifying missing variables is just a simple example, but more comples features can be transferred.
45	Line 322: similarly, you need more here than just "is important". For what species? What does the relationship look like?
	Reworded: "The inclusion of this abiotic variable, which was deemed second in importance just after the annual precipitation, by the Featura Importance method..."
46	Line 355-57: what evidence is there for this statement? Also, how would this explain anything about how the two stage model works? The two stage model is trained on the ACTUAL numbers that were observed, it's not magically cooking up some potential population size prior to competition, as no such data exists (all predictions are trained on post-competition data).
	We reduced the specualtion on this paragraph. However, we kept the idea that the first step of the model predicts potential abundance based on abiotic variables.
47	Line 362: There's no evidence to back up this assertion as the model was in no way trained on size-structured data. There's no way the model could be making inferences about size-variation.
	We removed that sentence.
48	Line 363: When you say "as expected", where was this expectation layed out. I don't remember any hypothesis that Random Forest would outperform XGboost.
	Fixed, it was not a valid expectation.
49	Line 364: see previous comment about linear regression being a bit of a straw man as actual composition analyses are going to use more advanced methods (CCA, Mantel tests, etc)
	We clarified this along the manuscript, and mention explicitely other alternatives and what is our two step model contribution over existing methods.
50	Line 366: yes, in theory data-driven models could enhance mechanistic models, but it's one thing to say it and another to do it. There were no such models in the Intro, Methods, or Results.

We agree that we do not fully explore this feed-back, but we do provide a simple example on how our model help identify important variables not considered in mechanistich models. Further work can explote them to incoportate functional forms or interactions between variables. This is emphasized now in text.

51	Line 373 with not wit
	Fixed
52	Line 378: It's inappropriate to introduce a completely new analysis in the Discussion without any prior mention of this in the Intro, Method, or Results. Furthermore, insufficient information is provided about how the model was actually fit, what the parameter estimates are, what the model actually predicts for each species, and we learned from this model. Finally, I don't really see a good argument about how machine learning actually helped here in improving mechanistic models. This model is completely parameteric (which I'm fine with) and sure you tried adding a covariate to it based on what you learned in the ML model, but you also could have tried adding your covariates to the model without having done the ML analysis first. And nothing about the shape or nature of the responses was at all informed by the ML analysis. This would be a very different argument if the authors had used the ML analysis to propose functional forms that they wouldn't have proposed if the ML analysis hadn't shown them the (approximate) shape of the relationship in a way that wasn't readily apparent (e.g. from just making simple scatterplots of abundance vs each covariate, or d(abundance)/dt vs each covariate)
	The reviewer is completly right, and we state this as future work clearly in text.
53	Line 382: in not int
	Fixed
54	Line 396: drop "we are confident it will be likely to obtain models that accurately predict temporal variation in species abundances". It is pure speculation that the temporal models will be accurate, and adding the "likely" makes the statement meaningless.
	Fixed
55	Line 408: These models are by no means simple (40 predictors) and scale just as poorly with number of species as the models in the Intro that you (rightly) pointed out don't scale. For example, right now you have to fit 40 terms for each of 23 species (920 terms fit to 45 site-years of data). If the community was twice as diverse you'd then need to fit 63 terms for each of 46 species (2898 terms).
	We changed it to "Reliable" instead of "simple".
56	Supplement 1: I'm not following what the logic is behind the statement "The error associated to the predictions from the population dynamics model is not directly comparable to the error associated to the data-driven model, because of the different methodologies and projections;". You are predicting the same quantities (Xj) for the same plots in the same years, so I don't see any reason you can't calculate the same error statistics and ask which model does better at predicting the temporal dynamics.
	The remark is right and we have removed that sentence
57	Also, for supplement 1 equations 1 and 2, if you substitute 2 into 1 you can simplify terms to a + b *exp(…). This means that the original model would be statistically nonidentifiable (which is why I asked earlier about how the model was fit) and is likely to make better predictions in this reduced form.
	We apologize for the lack of clarity on this particular analysis. The terms g_i and s_i are constants, and the terms fitted are the lambdas and the alphas. As such, the model is actually of the form mentioned by the reviewer. This is now explicited in the Appendix. The details of the maximum-likelihood fitting process can be consulted in the study by Garcia-Callejas et al. (2020) and their associated R package: https://github.com/RadicalCommEcol/cx

REVIEWER #2

#	Remark
General	This paper proposes a two-step ensemble model and feature engineering workflow to predict species abundances in real world populations over space and time. The aim is ambitious, and it speaks to an important division in quantitative ecology between traditional mechanistic population models, mostly using formal Bayesian models with MCMC, and more flexible machine learning models. The work is similar to semi-supervised learning with weak labels where the predictions from the first step are propagated to avoid measuring difficult interaction covariates in the field. They provide a nice worked example. I like the idea and think the conversational tone throughout is useful. Given that formal models have been largely non-effective, and that there is no hope is directly measuring species interaction coefficients over long periods of time, we definitely need all new ideas we can get. To make this paper more palatable to a large audience, the introduction needs to slow down, avoid making grand statements, and really consider the current state of quantitative ecology. My only concern for the results is that with a single example, and no simulations, it is difficult to guess which parts of the model will be most sensitive to typically challenging ecological scenarios. A short list might include<br><br>• Incomplete detection<br>• Large clines in environmental conditions<br>• Spatial autocorrelation<br>• Rare events/time lag (such as seed back in this case)<br>• Increasing numbers of interacting species<br>• Asymmetric interactions<br><br>It would be impossible for the authors to tackle all of these ideas, but without alteast some exploration in the text, I don't know that I see this as more than a nice example and perhaps not broad enough for the journal. I think this can be fairly easily remedied. The authors might feel the natural desire to defend their ideas and avoid being too critical, but I prefer that when a new modeling approach is proposed that we can assess both its strengths and weaknesses. The paper lacks clear guidance and when such a workflow is a good idea and when it would be problematic and rather opts for statements like L354<br><br>'The fact that this two-step process enhances predictions over a one-step model with all data available is remarkable'
	Thank you for your positive assement. We agree that while the two-step process proposed is easely extrapolable to other questions involving species interactions, its performance is still unknown and will depend on factors such as number in interacting species, detection, etc... We are all for providing a honest picture and hence we included a paragraph deliniating future work in the discusion. In addition, we tested for spatial autocorrelation in the paper as suggested also by reviewer 1 (please, see response to remak #1, reviewer #1).

1	The authors are really taking on a large and important moment in quantitative community ecology. Decades of mechanistic models show relatively little promise in predicting population abundances dynamics in natural settings over broad spatial areas. These conditions need to made clear in the paper, there are plenty of very good community microcosm experiments (https://scholar.google.com/citations?hl=en&user=Nkgv64gAAAAJ&view_op=list_works&sortby=pubdate). See several papers by H. Lynch in predicting penguin population dynamics (particularly https://besjournals.onlinelibrary.wiley.com/doi/full/10.1111/1365-2656.12790) or very similar work by J. HillrisLambers (https://onlinelibrary.wiley.com/doi/abs/10.1111/ele.13236) showing that current models can't produce large scale dynamics. I'll avoid citing my own work here, but there are many similar overarching papers outlining the need for prediction in community ecology [1–3]. I don't think the paper needs to repeat the work in those papers, but it needs to help set the stage. Rather than creating a simple old/new dichotomy between mechanistic and data-driven models (L24), I think the authors need to spend more time in setting up the tradeoffs between the two. Why were mechanistic models originally favored? Data availability, lack of computational power to do MCMC? Focus on hypothesis testing over prediction (see [4])? To make the kind of impact they want, the authors need to slow down in the intro, give some specific examples, rather than general citations, and really engage with the current state of the field. Try to avoid proposing a 'solution' (always dangerous, L22) rather quickly. I'm a very sympathetic reader, since I already use these kinds of models, but the target audience should be a much broader group of quantitative ecologists, so the arguments need to be well laid out.
	We really thank the reviewer for this point, and we totally agree on the importance of using both approaches depending on your aims. We tried to incorporate this thoughts through the introduction and discussion.
2	L51. I work with these kinds of ML ecological models every day. I emphatically reject they have 'solved' anything (famously see [5]). I could show some well-constructed and thoughtful neural networks that do an absolutely terrible job at prediction compared to regression. Similar to the comment above, if the authors want to really make a contribution in this area, more subtly and accuracy is needed. L55 is particularly worrisome and shows some CS envy. This kind of sentence makes me stop reading a paper.
	We reworded the paragraph to provide a more balanced view. It was not our intention to discredit any other approach
3	One thing that jumps out at me is that the virtue of the sequential ML models, especially in the computer vision space, has been their ability to scale to enormous amounts of data (going back to [6]). The argument has always been that the learning potential for these types of models greatly exceeds the formal hierarchical Bayesian models and does away with needing to set priors. But in ecology we almost never have such large datasets (though its getting easier). I worry about the applicability of this kind of method to the average ecology dataset, especially as the number of species increases. How would this scale? I don't have an intuition for which parts of the model would be most sensitive to larger number of taxa. Similarly for spatial heterogeneity of the landscape. The authors should either dial back their language in the conclusion or look to simulations to help explore when these kind of models will be most successful.
	We provide now examples on when this approach will be useful (i.e. in combination with ongoing monitoring schemes), and what needs to be further validated to upscale this type of analysis.
4	Figure 2 B needs some text to help orient me. What do the authors hope to convey with this right-hand panel?
	The right-hand panel is a depiction of the distributions of RMSE values for each of 100 experiments of ABIOTIC, ALL FEATURES and TWO STEP models. With the new results histograms overlapping is reduced and we hope the plot is now more legible

5	Slightly awkward phrasing in the abstract ‘inform back’. In general, I try not to provide too many line edits unless they interfere with readability. Missing ‘e’ in ‘especially’, in the abstract.
	Reworded: "In addition, we show that these data-driven models can also suggest how to improve mechanistic models adding missing variables that affect species performance such as particular soil conditions"
6	Try to be careful in the introduction to consider a wide range of sources. The topic is heated and will be best received if the authors don’t cite their own work in such prominent spots (e.g. L7-11).<br><br>1. Anderegg LDL, HilleRisLambers J. Local range boundaries vs. large-scale trade-offs: climatic and competitive constraints on tree growth. Ecol Lett. 2019;22: 787–796. doi:https://doi.org/10.1111/ele.13236<br>2. Youngflesh C, Jenouvrier S, Hinke JT, DuBois L, Leger JS, Trivelpiece WZ, et al. Rethinking “normal”: The role of stochasticity in the phenology of a synchronously breeding seabird. J Anim Ecol. 2018;87: 682–690. doi:https://doi.org/10.1111/1365-2656.12790<br>3. Dietze MC, Fox A, Beck-Johnson LM, Betancourt JL, Hooten MB, Jarnevich CS, et al. Iterative near-term ecological forecasting: Needs, opportunities, and challenges. Proc Natl Acad Sci. 2018;115: 1424–1432. doi:10.1073/pnas.1710231115<br>4. Betts MG, Hadley AS, Frey DW, Frey SJK, Gannon D, Harris SH, et al. When are hypotheses useful in ecology and evolution? Ecol Evol. n/a. doi:https://doi.org/10.1002/ece3.7365<br>5. Nguyen A, Yosinski J, Clune J. Deep Neural Networks Are Easily Fooled: High Confidence Predictions for Unrecognizable Images. 2015. pp. 427–436. Available: https://www.cv-foundation.org/openaccess/content_cvpr_2015/html/Nguyen_Deep_Neural_Networks_2015_CVPR_paper.html<br>6. Dean J, Corrado GS, Monga R, Chen K, Devin M, Le QV, et al. Large Scale Distributed Deep Networks. NIPS. 2012.<br><br>Reviewer #3: Kia ora koutou,
	Thanks for the refences. Citaions has been fixed as suggested.

REVIEWER #3

Remark

I wonder how much the two step model performance depends on the strength of the interaction. One way of understanding this, would be to compare the expected size of the population in isolation vs accounting for interactions. How quickly the method would stop to be accurate if the interactions increases?

This is a good point. We know competitive interactions in our study system are important (see Lanuza et al 2018 cited in text). Note that the "abiotic model" ignores the abundances of competitors species, and hence, assumes interaction strength equals zero. In this situation, the performance is remarkably worse than when interactions are accounted for. We address this point in the discusion.

How would you account for other kind of ecological interactions (plantpollinator, plant-herbivore, . . .)? If we are considering the method in the framework of climate change adaption, may the impact of climate change on other guilds of the ecosystem be an even stronger force than abiotic ones?

We agree that other interactions are important for determining population sizes. When those are known, they can be added as extra biotic variables, but unfortunately this is rarely the case. In addition, plant-pollintor and plant-hervibore are often determined by the plant's abundance, hence, by modelling plant abundance our model may be indirectly capturing part of this variablilty. We address now this issue in discusion.

It is not clear to me the dependency from the spatial and temporal model.
In particular, I can’t understand how the “latter” temporal model is different from the spatial model. It may be worth to explicit precisely what data is used at each step of training and testing for each model.

The section "The problem" has been rewritten to explain the differences of spatial and temporal applications of the predictive model.

I would not show RSE here as, without a context, it doesn’t mean much (at least to me: it’s a big number? a small number? what would be a big number?).

RSE figure has been removed from abstract, following the suggestions of Reviewers #1 and #3

The motivation for the paper is clearly expressed, however the mathematical formalisation of it is unnecessary heavy.
Taking for example Eqn (1), I would suggest to rewrite it as:
~X_j (t) = g (A_1 (t) , . . . ,A_n (t) ,X_1 (t) , . . . ,X_n (t))
That is: the term X_2 (t) is unnecessary and the dependency on the subplot k can be stated once and for all (and then drop from notation). This holds for all the equations in this section.

We simplify the notation as suggested, dropping k from the notation

In Eqn. (2) the subplot becomes m instead of k. I couldn’t understand why, and it conflicts with the m stating the number of species. In Eqn. (5) the subplot becomes p. Keep k everywhere (and keep it implicit for a lighter notation). In Eqn. (5) I would consider using ~g instead of f as f has the same arguments and outputs of g and it is indeed the task of the machine learning models to approximate it. This also makes clear that there are two approximation steps, one for ~X and one for ~g.

Fixed following the reviewer's advice. Equation 2 is dropped because it was redundant and confusing, we reword the sentence to explain that is the application of the predictive model to one of the sampled subplots whose data wehere not used to build the model. Eq 5 is rewritten as suggested, as well.

Also, when introducing the two step spatial model (based on Eqn. (5)) I got at first confused about the temporality of the events being predicted at each step.
My current understanding is that: - Step (1): from the A(t − 1) and X(t − 1) predict A_pred(t) and ~X (t) (the abundances if there were no other species in the plot) - Step (2): from A_pred(t) and ~X(t) predict “biotic constrained abundances” X(t) If that is the case, and I’m not confused, it may be worth to explicit it.

The wording was confusing, and there was a typo in former equation 4. In the first set ~X_j(t) abundances are predicted from A(t) values, that are easy to get. In the second, A(t) and ~X_j(t) are joined to get the ^X_m(t), a stronger prediction of abundance of the focal species m. If we need to predict abundances at instant t+1, we then need just the A(t+1) values that are easy to get. We can also predict abundances of a focal species at a different subplot at instant t, just knowing the A(t) values.

line 111: I would use the term ‘subplot’ instead of ‘area’ as the term ‘area’ is not used again throughout the text

Fixed

line 180: I would drop “Therefore”

Fixed

line 192: “black models” -> “black box models”

Fixed

lines 294 - 297: I’m not sure I would say that “machine learning” methods for time series prediction require, in general, a trend and a seasonality. Any machine learning method should, at least, predict quite well a constant times series (one that never changes value). Either or both of those things can be missing, and prediction can still be made. Yet, I may be misunderstanding what you are saying here.

The paragraph has been reworded and simplified. The observation about trend and seasonality was a remark about classical time series prediction but as they were discarded in an early stage of the research the explanation is pointless

lines 256 - 259: Maybe explicitly formulating the models for the spatial and temporal predictors might clarify the distinction between the 2 models for readers specially for the step 2? (It could be included in the supplementary information)

We have made several changes to explain that the model is unique and has two applications, spatial or temporal, depending on the selection of the training set. We have improved the description of the two-step method as well. (See remark #16 of reviwer #1)

Results from S1 sounds like some interesting results, would it be worth mentioning in the main body?

Now referenced in the first paragrah of the Results section.
