# Supplementary material for: Fine scale prediction of ecological community composition using a two-step sequential Machine Learning ensemble
Source: PLoS Comput Biol. 2021 Dec 6;17(12):e1008906. doi: 10.1371/journal.pcbi.1008906 (PMC8675934; doi:10.1371/journal.pcbi.1008906)
Supplement: Attachment — Submitted filename: response_to_the_reviewers_2nd_revision.pdf [file pcbi.1008906.s005.pdf]

Reviewer #1

Introduction: At a high-level, I'm still concerned that the authors are couching their paper in terms of the (real) scaling problems inherent in theory-based modeling approaches to species interactions (e.g. Lotka-Volterra; Lines 21, 25-48, etc), but then glosses over that (A) the method they propose has the exact same issue (number of terms scales quadratically with the number of species and (B) other mechanistic modeling frameworks exist that don't have this scaling issue. See previous Comments XX.

We believe our methodology does not incur the problem of the quadratic increase of model parameters with the number of species. We use information of species interactions to see whether this data improves model performance and actually it does not, if we select the two-step best model prediction. It is true however that our models can be data hungry because we are trying to predict a high-dimensional problem, which is fine scale prediction of species abundances at the $1m^2$ level of more than 20 species. However, our model does not rely on having information of species interactions, which we agree inherently scales quadratically for any kind of model.

Line 56: The authors point to the lack of interpretability of machine learning models, but then go on to present an uninterpretable analysis. For example, there are still no plots showing the shapes of the relationships inferred (previous Comment XX). The two-stage modeling approach is also particularly hard to interpret because it's not clear what these stages represent. Counter to what is implied (but not outright stated, e.g. Line 71 and 406), the ABIOTIC model is most definitely NOT a model of the fundamental niche, but instead is a model of the realized niche, with the impacts of species interactions still implicitly present in the data.

Because Random Forest models are not mechanistic, we prefer to not overstate the idea that one step of the model represents the fundamental niche and another step of the model represents the realized niche. We provide evidence that the Random Forest models presented here are good candidates to predict fine scale spatial species abundances, but we also agree they are disconnected from previous modeling approaches. They could be seen as a completely different approach to model species abundances. Therefore, we have removed any reference to niche-based processes.

We have reworded and simplified the last paragraph of the introduction section:

"However, we can combine the power of data-driven models with stronger theoretical foundations. Here we address this issue by partnering together ecologists and data scientists to develop an efficient and predictive data-driven model rooted in known ecological mechanisms that are thought to explain species occurrence and its abundance at local scales."

Equation 2: It's not clear to me what point is being made here. The only difference between equation 1 and 2 is that you're calling the function g instead of f.

We wanted to stress that the first equation corresponds to a mechanistic model and the second to a data driven model for the same problem.

We add the following sentence after introducing g: "We call it so to make it clear that it does

not belong to the set of mechanistic models of equation 1."

Equation 3: As noted in previous Comment XX, I have real concerns about the approach to temporal that's implied here, which differs strongly from standard time-series and population modeling approaches (which is to use X_t to predict X_t+1). Indeed, in this round of review I downloaded the authors data and can confirm that, counter to their abysmal results in Table 3, a linear model that just use X_t to predict X_t+1 (without ANY other covariates) has an R2 of 0.42 (compared to the reported -3.95 to 0.08). Adding in species identity and the precip for both time t and t+1 (but not ANY soil variables, species interactions, interactions between species and precip, or nonlinear relationships), a simple linear model increases to a R2 of 0.56. Admittedly this is still worse than the spatial Random Forest, but it does strongly suggest that the Authors revise their approach and all of their Results and Discussion around temporal models.

We believe that our choice of "temporal" adjective was not fortunate. We never tried to mean the prediction of abundances of X(t+1) based in X(t), but the prediction of X(t+1) for one of the sampled subplots using the full dataset data. In the original formulation, a subindex was used to denote the subplot, but it was removed following a reviewer's suggestion. Hence, the spatial and temporal models only differ on the data used to train and test the model.

To avoid this misunderstanding, we have changed the manuscript to refer to "temporal trained" model.

"For instance, if the set includes a long enough series of data recorded during previous seasons, you can train the model with that set and predict the abundances at subplot during season t+ 1, given that all the covariates are available, and that subplot k belongs to the sampled set. That is what we call the temporal trained predictive model. Note that we do not try to use a time-series approach.". There are more minor scattered modifications due to this change.

The observation about the poor performance of the temporal trained RF compared to a simple linear model is fair, but our goal was to stress the importance of data availability and the striking difference of performance for different applications of a same curated set.

We add the following paragraphs in the Methods [1] and Discussion [2] sections:

1- "We do not explore here other approaches such as the use of time-series data."

2- "For this last practical purpose an alternative approach based on time-series may yield better results."

As a separate comment, accessing the raw data made it clear to me that the soil covariate data for a subplot were identical for every single year. First, this was not obvious to me when reading the paper so should be more prominent in the Methods. Second, it should be reiterated in the Results and Discussion, as it's utterly unsurprising that their current Temporal model does so poorly when the covariate data used to predict Temporal variations is itself not changing in time.

In the data description, we included the following description "In addition, we empirically measured at the subplot level an array of physical and chemical soil properties at the

beginning of the survey (spring 2015) to characterize the abiotic properties of each community (one soil sample per each subplot, see Table S2 for a summary of the soil properties obtained)."

We add the following statement in the Data description subsection:

"Soil data was only recorded during 2015 because these are soils with high content of clay in which their properties vary little from one year to another. Therefore, although we acknowledge that within the five years of study some soil properties might have changed, we assume this variation is of little magnitude."

And we add this sentence to Discussion as well. "Including the temporal resolution of soil properties may enhance model performance".

Line 118: measuring, not measure

Fixed

Line 134: Unless you have a time machine I don't know about, measuring abiotic data for time t+1 when you're currently at time t isn't possible. If you want to predict to time t+1 you need to either use covariates at time t or (uncertain) forecasts of covariates to time t+1. Second point, throughout the paper (here, L147, etc.), the authors keep saying that it's easier to measure abiotic covariates than it is to measure the biotic data itself (species composition) but they don't provide any evidence to support this argument. Indeed, as previously noted their own data set samples species every year, but abiotic covariates only once, strongly implying that the latter is harder. On top of this, there are known technological approaches to measuring species composition at fine spatial resolutions and non-trivial spatial scales (e.g. NEON's 1 m^2 resolution imaging spectroscopy), but as far as I know there is no equivalent technological approach to measuring below-ground nutrient concentrations.

Abiotic data was easier to obtain than biotic data of species interactions, but we did not follow those measures as they were not the initial focus of the project. We focused on the biotic component as this was the kind of information nobody else had. It is also worth noting that there are variables that vary over years such as those related to climatic variation (temperature and precipitation). We clarified this in text. We also cited NEON in the beginning of the discussion section:

"The rate of ecological data generated is increasing substantially [63]."

Line 150: I'm not sure why the authors think this approach is "too radical" since they're just describing species distribution modeling, which is widespread in the discipline.

We reword the sentence: "From an ecological perspective this model ignores direct species interactions"

Line 166: Unclear how the uncertainties in the predicted values are being handled in the second stage model. Please comment on this explicitly. I suspect they are being ignored, in which case this should be revisited in the Discussion as a limitation / future direction.

For the two-step model, it must be considered that during the first stage the abundances of

each competing species are calculated based on abiotic parameters. This can be a direct estimate in the case of more common species or more unique edaphic conditions. However, when we find species that are not so abundant (median value of individuals equal or less than 3) uncertainty may arise during the first predictive phase. This fact gives rise to specific errors in the performance of the model.

As a future direction, it is possible to correct this fact by oversampling these singularities in the dataset, so that the model is able to learn the particularities of each species within a subplot. However, this can be considered a delicate technique as it can lead to overfitting. Given that in this analysis the cases of uncertainty due to this fact were not very common we discarded this option.

We add the following paragraph in the Discussion section:

"When the abundance of individuals is low (median value under 3), the uncertainty of the abiotic prediction increases. To avoid this issue, the model could be refined in a future development through careful resampling of low abundance species before performing the first step".

L198: still not adequately addressing how the approached described deal with the zero inflation problem (previous Comment 2)

When the prediction is performed with all covariates, zero inflation is not hard to deal with, as far as the proportion of abundances of the training set does not deviate from the original distribution. However, when using the two-step model, it could happen that for the second step we could face a dataset with hardly any abundances for some species. In this case, it would be necessary to address oversampling techniques. We tried that procedure, but the overall improvement in R2 was low unless the oversample percentage was extreme (about 500% for low abundance species). We discarded that possible improvement path, and so the values of R2 are poorer for that set of species (figure 3)

We add the following sentence in the Discussion section: "We discarded this procedure because to raise the overall R2 with simple SMOTE-based resampling required too high resampling percentages for this particular application [61]"

Line 304-305: sentence very hard to parse. I had to read it 3-4 times just to be sure it was a sentence, not a fragment, and it was still unclear what the authors were trying to convey.

Once we drop the correlated abiotic variables, we run the Random Forest Feature Importance method with this reduced abiotic set (Table 1) and the full set of features (reduced abiotic + competitor abundances, Supplementary table S4).

We have reworded the paragraph:

"After we drop these variables we run the Feature Importance method for the Random Forest with the abiotic set (Table 1). Results show that Annual precipitation is the most relevant abiotic feature after Species, that is just the focal species whose abundance we want to predict. Carbonates, C, P, and Salinity follow in importance, while Ca, Mg and pH are less relevant than the added random noise, so they could be ignored to build the final model.

We apply the Feature Importance method with the full set of features as well (Supplementary

Table S4). Results show that Annual precipitation is, again, the most relevant abiotic feature. The number of individuals of abundant competitor species such as POMA, LEMA, CHFU, and SASO (see Supplementary Table S3 for species acronyms) or the concentration of carbonates showed up to be relevant too for the Random Forest built with the full set."

Line 311: Not entirely following why this was done over just not including species j as a covariate when predicting species j.

We build a unique model for all species. The focal species is one of the input variables of the predictor. We can't get rid of the column of the number of individuals of focal species as competitors, because that feature is necessary to train the model when the focal species is different.

In order to safeguard this problem a value 0 is assigned before building the model, no matter if we are working with the ALL FEATURES predictor (with competitor abundances recorded in the field) or the TWO STEP (with competitor abundances predicted with ABIOTIC features).

We have reworded the paragraph:

"As the model is unique, there is a circular problem with the abundance of individuals of the focal species when acting as competitors. For instance, to predict the abundance of HOMA individuals in a particular subplot, we should know in advance the abundance of HOMA individuals as competitors. Getting rid of the HOMA column is unfeasible because those values are important to predict the abundance of any other species. So, before building the full set model, this value is set to 0 where the competitor and focal species are the same. "

Line 322: latter not former

Fixed

Figure 3: The decision to both use a different species order in panel B, and to switch the mapping between color and species, is extremely misleading. I think it is essential to keep the colors the same between panels and recommend keeping the species order the same too.

Figure 3 is modified as suggested.

Line 356: why? Please revisit in Discussion

The existence of competing species seems to play an important role as an inhibitor of the growth of a particular species. This information is lost when the model only works with abiotic features. The addition of predicted abundances of competitors has a positive influence on the estimation of absences. This is just an observation, not an explanation, so we reword the sentence as "The two-step Random Forest model seems to be much more accurate predicting zeros than the abiotic RF model" and include the following text in the Discussion:

"A remarkable fact is that the two-step model is much better at predicting absences than the abiotic one. The existence of competing species seems to play an important role as an inhibitor of the growth of a particular species. This information is lost when the model only works with abiotic features."

Line 369: First, this comes as a surprise to me as I would have expected a simple repeated measures model to have done fairly well because of the tendency of plants to stay

put through time. Second, as discussed above I don't think this analysis is correct.
We included this paragraph in the Results section:<br><br>"This annual plant system is a highly variable system in which propagules can disperse over a wide range of distances after individuals complete their life cycle. Such dispersal kernels in combination with variation in flooding events make our system overall highly dynamical in terms of space and time. We therefore do not believe that our system is stable through time as it can be other systems with species with longer life cycles such as shrubs or trees. "
L377: ambiguous what "this variable" is referring to.
Reworded as: "One possible workaround is dropping the annual precipitation to reduce overfitting."
L391: Feature
Fixed
Line 392: Still introducing new Methods in the Results. This is not OK. Per previous Comment 8, you really need to lay out this analysis in the Methods and preferably also motivate it in the Introduction.
We expanded the methods to reflect all this steps. In addition, we add this sentence in the Results section after Table 3<br><br>The inclusion of this abiotic variable, which was deemed second in importance just after the annual precipitation, by the Feature Importance method (Table 1), shows an overall improvement in the predictions derived from the mechanistic models (S1 Appendix).
Line 444: This likewise feels like new Methods and Results in the Discussion. Mechanistic modeling part not adequately explained.
We expanded the methods to reflect all this steps. In addition, we add this remark in the Discussion section:<br><br>"With this new knowledge, we re-parameterized the mechanistic annual plant model by adding $CaCO_3$ as a covariable affecting both the intrinsic fecundity rates and the pairwise interactions among species."
Comment 5: I'll defer to the Editor, but I'm not fully convinced that a comparison to conventional methods for vegetation analysis (e.g. CCA) is "beyond the scope". Seems like at a minimum the existence of such methods should come up in the Discussion.
We added a section in discussion about alternative modeling approaches and reinforced the idea that our approach is complementary, and not necessarily a substitute from other approaches:<br><br>"We want to highlight that the proposed approach complements current approaches to understand fine scale community composition, such as multivariate methods (e.g. CCA [65]) or time series analysis [66], which may be more suitable depending on the question

to be answered, or the data available. ”

Comment 6: did not address the issue about differences in sensitivity to spatial and temporal anomalies. Would be good to add a quick line to the Discussion.

We believe the sensitivity of our model to perform better in terms of space than time is due to the sampling size; we have 324 plots of measurements for one years but only 5 years of data. To explain these different sensitivities we have reworded lines 408-410 as: “Our models are sensitive to the breadth of the training data, and as such they capture better the spatial anomalies (where we have more data) than the temporal anomalies”.

Reviewer #2

I appreciate the authors willingness to engage with reviewers and improve the text. The intro is much better. I like the idea and think it is worth exploring. The authors continue to overstate the strength of their results, but I think this point is stylistic and rather minor.

We deeply appreciate the reviewer for the fruitful discussion at work thanks to the questions and suggested changes, as well as the time taken to review our article. We have tried to reduce any exaggeration of the results on paper.

Reviewer #3

Thank you so much for you effort in answering my questions.
I'm satisfied with the answer and the edits.

We thank the reviewer for helping to improve the paper.
