# Supplementary material for: Fine scale prediction of ecological community composition using a two-step sequential Machine Learning ensemble
Source: PLoS Comput Biol. 2021 Dec 6;17(12):e1008906. doi: 10.1371/journal.pcbi.1008906 (PMC8675934; doi:10.1371/journal.pcbi.1008906)
Supplement: Attachment — Submitted filename: Civantos2021PLOSCompBio.docx [file pcbi.1008906.s002.docx]

Civantos-Gomez 2021 PLOS Comp Bio

“Fine scale prediction of ecological community composition using a two-step sequential machine learning ensemble”

In this paper the authors use a two-stage machine learning approach to analyze and predict species abundance data for a Mediterranean grassland as a function of precipitation, soil properties, and the predicted abundances of the other species in the system. At a high-level, I can see the intuitive appeal of the two-stage model, but the authors haven't explained the ecology of whether this approach makes sense or not, nor have they explained how the approach actually works (e.g. aren’t you just using the same data twice? And can’t you just substitute one model into the other to end up with a model you could have fit non-sequentially).

Beyond this conceptual question, I have a lot of concerns about how the analysis itself was actually carried out:

Spatial correlation and sample size: How is spatial autocorrelation among the 36 subplots accounted for? What about the 9 plots (which are all < 1km apart). Abiotic properties are measured at the scale of the plot not subplot (line 158) so how is this not pseudoreplication? We really need to see a variogram or correlogram showing a lack of spatial autocorrelation (which, honestly, is a bit unfathomable) otherwise you need info about how spatial correlation structure was included in your models. In addition, if you are relying on ensemble resampling (in fitting) and cross-validation (in validation) is absolutely critical that you do so blockwise (i.e. dropping whole sites) otherwise your error statistics are going to be massively optimistic.

Zero inflation: The dataset being analyzed contains 75% zeros, but the authors don’t really explain how they handle the distinction between presence/absence and abundance. They are working with models that don’t have built-in range restrictions (abundance can’t be negative), nor do they account for the discrete nature of abundance data, but they say nothing about how this affects prediction (e.g. models could easily predict non-sensical negative values, but we don’t hear/see anything about how often this occurs or how it’s handled; see Model Performance below) or scoring (are RMSE and RSE really the right metrics for scoring zero-inflated count data?)

Model selection: Counter to the basic concept of parsimony and decades of literature on model selection (plus just common sense), the authors retained covariates that contributed less to the model than random noise. Similarly, covariates were included in the model that were massively colinear, counter to what anyone would learn in a basic stats course, even when it’s obvious a priori that these are not telling us different things (e.g. including all of C, N, C:N, and organic matter; including all of sodium, chloride, and salinity).

Model performance: There are no plots showing how the model actually does at predicting abundances, just plots of error statistics. Similarly, there are no plots of what the inferred relationships between inputs and abundance actually look like, which severely limits interpretability and greatly reduces what we’re learning from the analysis. There’s not even information on a species-by-species basis about the relative importance of the different abiotic covariates and where different species are sorting out on the abiotic gradient. Similarly, there’s no information anywhere in the paper about what the ML model tells us about how the species are interacting (just a correlation matrix of the RAW data)

Strawman comparison: In the paper the authors compare their ML models to predictions made by a linear regression. I think this is a strawman comparison because conventional analyses for community composition data have for many decades acknowledged that the relationships between predictors and abundance are nonlinear (e.g. Canonical Correspondence Analysis assumes a unimodal Gaussian kernel). In showing they do better than a regression, and then identifying the relative importance of a few abiotic variables, I don’t think the authors demonstrate that they learned something that wouldn’t have come out of conventional vegetation analyses.

Temporal model: Temporal model is poorly described, and in particular how spatial information is leveraged when making temporal predictions. Are the sensitivities assumed to be the same? That seems to be implied, and a space-for-time substitution may be OK as an initial hypothesis (in which case you may not need longer time series), but there’s also lots of evidence that responses to mean climate and responses to temporal anomalies need to be treated differently (though the two may interact).

Paper has numerous spelling and grammar errors; would really benefit from a copy editor. Mathematical notation is inconsistent.

The paper has new analyses showing up in the Results and Discussion that weren’t motivated or explained in the Introduction and Methods (e.g. fitting a simple demographic model), as well as analyses in the Methods that don’t have Results or Discussion (e.g. Taylors Law).

Detailed Comments:

Abstract line 9: I don’t think you benefit from having the word “deterministic” here, it’s just going to be a distracting source of contention (immediately caused the reader to start debating whether ecological processes are deterministic, which is beside the point [especially if they are predictable, which isn’t the same thing as deterministic])

Abstract line 14: RSE referenced for the first time without defining the acronym. This metric isn’t particularly common, so you can’t assume meaning from context, nor is it clear from context whether 0.13 is a good score). You might try using R^2^ instead, which is more commonly understood.

Abstract line 19: not clear what “back” refers to here

Abstract line 22: probably need a “predicting” between “at fine-scale”, as well as a comma before the “while” and the “can” on the following line.

Abstract line 23: especially, not specially

Author summary line 8: need a “by” in “abundances training”

Line 2: drop “different drivers of”

Line 8: “experienced fast growth during the last decades due to”

Line 27: missing a word in “require to feed” (e.g. require data to feed)

Line 46: the statement “high level of abstraction of mechanistic models” seems like an oxymoron to me (if it’s mechanistic, it’s not an abstraction). More generally, throughout this discussion you need to be much more clear about what you consider a "mechanistic model". Many of the statements you make apply to simple theoretical models (e.g. Lotka-Volterra), but I would not consider such models to be mechanistic, especially compared to the many plant community and population modeling approaches that explicitly account for demographic processes (e.g. matrix models, integral-projection models, individual based models, stochastic simulators like forest gap models) or ecophysiologically-based vegetation demographic models (see review by Fisher et al 2018 GCB). It is worth noting that while many of these approaches do have large numbers of parameters per species (or PFT), many also model resource competition explicitly (rather than having theoretical “interaction terms” and thus the number of parameters scales linearly with the number of species in the community).

Line 99: you should NOT include “individuals of Xj(k,t)” as a covariate when trying to predict the abundance of species j. Such a model is circular and trivial. The “including” should be “excluding”.

Line 115: no clue what “abundance of the plants that will ripe next year” is referring to. You’re discussing models of abundance, not maturation or fecundity.

Eqn 3: The model presented here is subtlety but importantly different from conventional time-series and dynamic modelling approaches to predicting temporal variability, which would use the abundance of the con- and heterospecific individuals at time t (not t+1) to make predictions to time t+1. The whole problem described in the rest of the paragraph changes dramatically when you do this. Yes, you still are faced with the problem of needing to know the current state of the vegetation (a.k.a initial conditions) at new locations, but that’s the same problem as your spatial prediction problem (not a new problem), and also one that is more constrainable by data than the “unavailable by definition” problem of knowing the abundances in the future. At the same time you may genuinely want/need to include the covariates at t+1, and yes those need to be predicted, but this is a standard ecological forecasting problem and not one that needs to be built into your ML model (e.g., using the long-range seasonal precipitation outlook to predict species responses to interannual climate variability). Finally, when making community-level predictions you wouldn’t be writing a separate model for each species, but would be writing a multivariate model that predicts a vector of abundances and accounts for the covariances across species.

Line 126: I can’t figure out how you would end up with 23 unsolvable problems when you have 20 competitors.

Line 148: Is “Prediction” the best title for this subsection? Seems like it’s mostly a description of the sites and data

Line 149-157: This is insufficient information about the field research. Where exactly were these plots (lat/lon? Country? Field station?). How exactly where the communities surveyed? How many soil cores were taken per plot? How deep? What methods/protocols were used to analyze the soil properties? What equipment was used? What type of system measured precipitation?

Line 165: When you say “Removing them”, what specifically are you removing? Need more info about the specific criteria you used to remove species or sites, whether that was for the whole analysis or just a specific plot, and how sensitive you results are to your removal criteria. In general, subjective criteria for dropping datapoints makes me nervous.

Line 167-171 and Fig 1B: Not sure why there’s this text and analysis about Taylor’s law. This doesn’t seem to be motivated by any question posed in the study (Intro), nor does it show up again in the Results or Discussion, it’s just a random analysis thrown in for no apparent reason. I’d recommend dropping.

Line 182: “generally works fine” feels like a subjective value judgement. Not sure why it’s here (rather than Discussion) nor why it’s relevant.

Line 198: If you are fitting a model with 40 covariates to a dataset that contains 5 years of data for 9 sites (45 site years) how is this not overfitting?

Line 200: organic matter

Line 204: what does “tried to” mean? Did the implementation fail somehow?

Line 208-212: First, the descriptions of these three methods is really insufficient to follow what is being discussed. Second, if you used the Filter method, why are you even discussing the Wrapper and Hybrid? No need to describe methods you didn’t use.

Line 213: First, the discussion of correlations seems like it’s a completely new topic, and thus should be a new paragraph (if it’s not a new topic, it’s unclear how it’s related to the filter method. Second, what is your threshold for “significant”. Third, how is it possible that there were no significant dependencies? The correlation matrix shows that C and OM have a correlation of 1! C also has a 0.93 correlation with n and 0.97 with C:N. How could these not be significant? Similar story with the correlations among salinity, Na, and Cl (not surprising). You absolutely should have reduced the dimensionality of these data before starting any analysis to avoid spurious results.

Line 215: This test is being introduced without any transition or explanation of what this is or why this is being applied.

Line 216: First, this is Results not Methods. Second, tell the reader which 25% was important (don’t make me stop and go find and interpret your table). Third and most important, were the other 75% of the variables dropped?

Line 220: this is also Results not Methods.

Line 223: The decision to retain variables that add less to your model than random noise makes absolutely no sense and is a pretty textbook example of what not to do in data analysis. Further, the argument that dropping the variables doesn’t improve predictions also completely misses the basic idea of parsimony and the core concepts underlying model selection. If we take the linear model as an example, every additional variable you retain increases the parameter uncertainty and thus increases the predictive variance. This is only worth doing if the residual error goes down by more than the parameter error goes up, such that the net predictive variance declines. Given that 75% of your variables are no better than random, adding them is clearly not improving the model, so they cause a net increase in predictive variance (which we’d see if the authors accounted for predictive uncertainty, which they don’t seem to be doing). But even adding a random variable will cause the residual error to at worst stay the same, and sometimes go down a bit by chance, so I have no idea where this “removal makes the model better” criteria is coming from.

Table 1: (1) Given that you described three modeling approaches, it’s unclear which model is being reported in this table. (2) what does “species” represent in the abiotic model? This factor wasn’t ever described in the Methods. (3) I’m assuming that this model is averaging the importance over all 23 species that were modeled, which seems like it could be really misleading, as we’re not learning anything about which species are associated with which drivers. (4) If C and OM have a correlation of 1, how come their importance scores are so different (with one being “significant” and the other being worse than random noise)

Line 228: Please don’t change your notation in the middle of the Methods as it’s really confusing. Up to now Xi were the observed values and \hat{Xi} was the model predictions. Now you’ve switch y for X for no reason. Also, much more confusing, you’re now using the hat for observations and the non-hat for predictions, which is the EXACT OPPOSITE of your previous notation.

Eqn 7: is there a reason to use RSE over the much more commonly used R^2^ (which should just be 1-RSE)

Line 252-253: First, it’s unclear why you would want to “avoid” the zeros in the data – the data is what the data is, and there’s no reason the expect absences to be “wrong” in any way. You should model the data appropriately (as zeros) rather than trying to transform your absences into something else. Second, the transformation you applied wasn’t described at all, you need to add a lot more detail. Third, given that the data is non-negative but you aren’t using distributions or models that enforce non-negative predictions, you also need to explain how you’re handling negative predictions from your models. Given a ~75% zero rate, I can only assume that there are a LOT of negative predictions. Fourth, while we’re on the topic, there’s not a single plot of what your model actually predicts in the whole paper (e.g. a predicted/observed plot, or plots of predicted abundance as a function of location, time, or your dominant covariates); how can a reader assess how your model is doing if we never get to see what it’s predicting?

Line 257: see previous comment about the need to block your training and cross-validation rather than subsampling randomly. See for example Roberts et al., 2017 “Cross-Validation Strategies for Data with Temporal, Spatial, Hierarchical, or Phylogenetic Structure.” Ecography.

Line 258: This is the only sentence in the whole Methods about the temporal model and the temporal predictions. You need a lot more info about what you actually did here.

General Methods comments: (1) What tools were used to do all of this? What versions of what packages and where are the citations? Right now none of these is reproducible. (2) your Methods never really explains how you’re handling the discrete nature of your count data, the zero inflation, or negative predictions. (3) given that you’re arguing for a sequential approach to modeling, it seems like you really need to show that your approach outperforms fitting the community data all at once (rather than sequentially).

Line 265: You should not be introducing new Methods details in the Results.

Line 269: I think you’re misinterpreting RSE, which is the % better than a simple null mean model, not the % error (which most readers will interpret as a something like a coefficient of variation)

Line 278: Three really important, bit points:
(1) There needs to be more information in the Discussion about WHY this sequential method improves prediction as what’s going on here is a bit too opaque and unintuitive and the explanations being given are too hand-wavy. To give a concrete example, if express the first abiotic model as \tilde{X} = alpha A (where alpha is a vector of coefficients and A is the matrix of covariates) and the second as X = beta A + gamma \tilda{X}, then you could just substitute \tilda{X} in to get X = beta A + gamma alpha A and it’s not clear (a) how this could possibly do better than just beta A [seems like it should be identical], (b) how it’s not overparameterized, and (c) where the additional information content is coming from to be able to improve predictions.

(2) If there's such a large improvement over a basic linear regression, that implies that key predictors are nonlinear. Please visualize those relationships so we can see if what the model is doing is sensible / biologically plausible or whether it's overfitting. This is also the ecologically interesting/important part of the model. Telling me that plants associate with soils and precip it trivial. Telling me what that relationship looks like lets me think much more deeply about what processes are at play here.

(3) Competing two ML models against a linear regression seems to be a bit of a straw-man argument. The long-standing textbook expectation for vegetation abundance along a gradient is that these patterns tend to be unimodal (e.g. classic papers by Whittaker from the 1950s). If abundance is unimodal, then the best-fit line will often be a pretty flat line (hence the trivial improvement over a null). This is also why things like Canonical Correspondence Analysis (which assumes a Gaussian abundance kernel) have been standard practice in community ecology for decades. This also reiterates my second point (we all already knew these relationships were nonlinear, but can you show that we really need something different from a Gaussian?)

Line 295: WHY would this be a required condition (seasonal variability and long term trend), especially if what your temporal model is trying to predict is interannual variability, not seasonal or trends?

Line 309-311: This point about variability falling outside previous observations reinforces my previous question about a lack of clarity of how spatial and temporal variability are being handled in these models. It seems less likely that the observed temporal variability fall outside the range of both the spatial gradient (n=9) and the interannual variability (n=5) simultaneously.

Line 312: I’m a bit concerned about the idea of dropping what is clearly the most important predictive variable (precip, table 1) unless you’ve also already dropped all the other less important variables (in which case you’re just fitting a constant mean)

Line 316-17: Be much more specific about what novel insights are coming out of these models. This needs to be something much more impressive than “precipitation structures communities” or “carbonates matter”, as this is too vague and also something that I suspect would have come out of traditional vegetation community analysis techniques (e.g. CCA)

Line 322: similarly, you need more here than just "is important". For what species? What does the relationship look like?

Line 355-57: what evidence is there for this statement? Also, how would this explain anything about how the two stage model works? The two stage model is trained on the ACTUAL numbers that were observed, it’s not magically cooking up some potential population size prior to competition, as no such data exists (all predictions are trained on post-competition data).

Line 362: There’s no evidence to back up this assertion as the model was in no way trained on size-structured data. There’s no way the model could be making inferences about size-variation.

Line 363: When you say “as expected”, where was this expectation layed out. I don’t remember any hypothesis that Random Forest would outperform XGboost.

Line 364: see previous comment about linear regression being a bit of a straw man as actual composition analyses are going to use more advanced methods (CCA, Mantel tests, etc)

Line 366: yes, in theory data-driven models could enhance mechanistic models, but it’s one thing to say it and another to do it. There were no such models in the Intro, Methods, or Results.

Line 373 with not wit

Line 378: It’s inappropriate to introduce a completely new analysis in the Discussion without any prior mention of this in the Intro, Method, or Results. Furthermore, insufficient information is provided about how the model was actually fit, what the parameter estimates are, what the model actually predicts for each species, and we learned from this model. Finally, I don’t really see a good argument about how machine learning actually helped here in improving mechanistic models. This model is completely parameteric (which I’m fine with) and sure you tried adding a covariate to it based on what you learned in the ML model, but you also could have tried adding your covariates to the model without having done the ML analysis first. And nothing about the shape or nature of the responses was at all informed by the ML analysis. This would be a very different argument if the authors had used the ML analysis to propose functional forms that they wouldn’t have proposed if the ML analysis hadn’t shown them the (approximate) shape of the relationship in a way that wasn’t readily apparent (e.g. from just making simple scatterplots of abundance vs each covariate, or d(abundance)/dt vs each covariate)

Line 382: in not int

Line 396: drop “we are confident it will be likely to obtain models that accurately predict temporal variation in species abundances”. It is pure speculation that the temporal models will be accurate, and adding the “likely” makes the statement meaningless.

Line 408: These models are by no means simple (40 predictors) and scale just as poorly with number of species as the models in the Intro that you (rightly) pointed out don’t scale. For example, right now you have to fit 40 terms for each of 23 species (920 terms fit to 45 site-years of data). If the community was twice as diverse you’d then need to fit 63 terms for each of 46 species (2898 terms).

Supplement 1: I’m not following what the logic is behind the statement “The error associated to the predictions from the population dynamics model is not directly comparable to the error associated to the data-driven model, because of the different methodologies and projections;”. You are predicting the same quantities (Xj) for the same plots in the same years, so I don’t see any reason you can’t calculate the same error statistics and ask which model does better at predicting the temporal dynamics.

Also, for supplement 1 equations 1 and 2, if you substitute 2 into 1 you can simplify terms to a + b *exp(…). This means that the original model would be statistically nonidentifiable (which is why I asked earlier about how the model was fit) and is likely to make better predictions in this reduced form.
